# Survey on Wireless Technology Trade-Offs for the Industrial Internet of Things

**DOI:** 10.3390/s20020488

**Published:** 2020-01-15

**Authors:** Amina Seferagić, Jeroen Famaey, Eli De Poorter, Jeroen Hoebeke

**Affiliations:** 1 IDLab, Department of Information Technology, Ghent University—imec, 9000 Ghent, Belgium; Eli.DePoorter@UGent.be (E.D.P.); Jeroen.Hoebeke@ugent.be (J.H.); 2 IDLab, Department of Computer Science, University of Antwerp—imec, 2000 Antwerp, Belgium; jeroen.famaey@uantwerpen.be

**Keywords:** Industrial Internet of Things (IIoT), LoRa, IEEE 802.11ah, WiFi HaLow, Time Slotted Channel Hopping (TSCH), Narrowband IoT (NB-IoT), Bluetooth Low Energy (BLE), BLE Long Range, WirelessHART, ISA100.11a

## Abstract

Aside from vast deployment cost reduction, Industrial Wireless Sensor and Actuator Networks (IWSAN) introduce a new level of industrial connectivity. Wireless connection of sensors and actuators in industrial environments not only enables wireless monitoring and actuation, it also enables coordination of production stages, connecting mobile robots and autonomous transport vehicles, as well as localization and tracking of assets. All these opportunities already inspired the development of many wireless technologies in an effort to fully enable Industry 4.0. However, different technologies significantly differ in performance and capabilities, none being capable of supporting all industrial use cases. When designing a network solution, one must be aware of the capabilities and the trade-offs that prospective technologies have. This paper evaluates the technologies potentially suitable for IWSAN solutions covering an entire industrial site with limited infrastructure cost and discusses their trade-offs in an effort to provide information for choosing the most suitable technology for the use case of interest. The comparative discussion presented in this paper aims to enable engineers to choose the most suitable wireless technology for their specific IWSAN deployment.

## 1. Introduction

**Figure 1:**
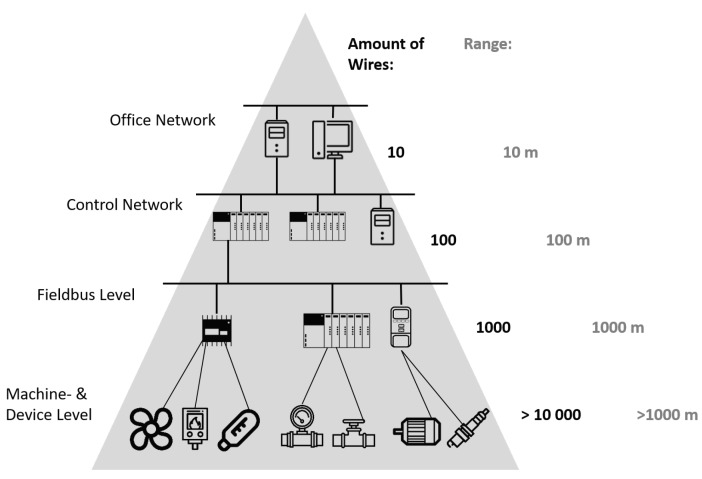
Node density exponentially increases from the top (office network/Internet/Intranet) to the bottom (machine- and device-level) in a typical automation system network hierarchy.

Industrial networks for process automation are deployed in sites which can be hundreds of meters wide, hosting very dense networks consisted of hundreds or thousands of nodes. Harsh industrial environments impose a number of challenges for wireless communications: reliability, fault-tolerance and low latency being the biggest ones. Unpredictable variations in temperature, humidity, vibrations and pressure make the industrial environments harsh, as well as the presence of highly reflective (metal) objects and electromagnetic noise. Even though not much data needs to be communicated in an industrial application, reliability and latency are critical, that is, delivery of all data must be guaranteed in real-time. Wired networks have met these requirements and are being used in spite of the high cost of wiring and the often present installation difficulties (see Figure 1) because wireless solutions are not as robust as their wired counterparts. Industrial automation systems in chemical industry, power plants, oil refineries or underground water supply systems implement complex monitoring and control processes. Thousands of devices send measured values (i.e., temperature, pressure, flow, position) to the actuators that control processes and to the servers that coordinate the production phases. Wiring is generally both challenging and costly (cca. 20 *$*/m): flammable, explosive and hot environments have to be avoided (e.g., in the presence of flammable gases in an oil refinery), remote or unavailable locations are hard to reach and mobile nodes can hardly be connected at all. Although wired networks at this time cannot fully be replaced by wireless networks in this domain, supervision and non-critical control with loose enough requirements could be realized over wireless. In addition, significant constrains that limit the practical deployments of wireless networks in such scenarios are battery capacity and power consumption of the devices. Ideally, communication and power cables can be mitigated to enable a fully wireless solution. For that, the devices should be energy efficient and able to power from a battery for years. Moreover, wireless networks introduce logical benefits that could be used in maintenance and commissioning, such as “plug-n-play” automation architectures to reduce downtime and speed-up tests and “hot-swapping” faulty modules. In addition to control and supervision, global wireless plant coverage could enable localization and tracking of parts in production, coordination of autonomous transport vehicles and mobile robots [1].

**Figure 2:**
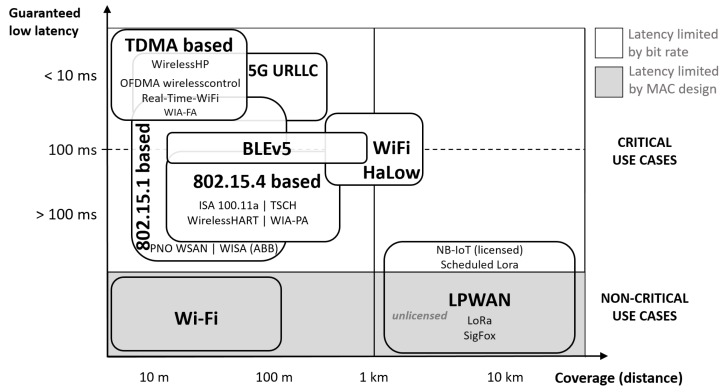
Different wireless technologies have different range/latency capabilities. This article discusses the trade-offs in mid-range technologies that could provide coverage of an entire industrial site (black boxes) with sufficiently low latency.

Industrial Wireless Sensor and Actuator Network (IWSAN) are gaining popularity in process industries due to their advantage in lowering infrastructure cost and deployment effort. The advent of Industry 4.0 already resulted in the successful use of IWSANs for monitoring applications and non-critical open-loop control in factory automation. A few new wireless technologies, such as WirelessHP [2], OFDMA wirelesscontrol [3], Real-Time-WiFi [4], Wireless network for Industrial Automation and Process Automation (WIA-PA) [5], can replace extensive wiring on industrial machinery, providing connectivity between machine parts with μs order of magnitude latency. Even though they enable reliable and fast communication, the range of such networks is limited to only a few meters, making them unsuitable for broad usage across an entire industrial site in process automation or for reaching remote areas if infrastructure cost has to be kept low. Ranges up to a few hundred meters are feasible with 802.15.4-based technologies such as WirelessHART [6], ISA 100.11a [7], 802.15.4g [8] with Time Slotted Channel Hopping (TSCH) [9] and WIA-PA [10], at the cost of other performance metrics. Sub-GHz wireless technologies, such as LoRa and SigFox, further extend the coverage due to the better signal propagation characteristics (up to 15 km and 50 km respectively) but are not suitable for frequent critical traffic given their low data rates (up to 50 kbps and 0.1 kbps respectively) which lead to very long transmission times in both uplink and downlink. In downlink, long transmission times also limit the gateway to serve many nodes, more so considering the duty cycle limitations [11]. Moreover, LoraWAN Class A and Sigfox only allow downlink transmissions that immediately follow uplink, resulting in substantial downlink delays due to buffering. NB-IoT experiences downlink delays due to buffering as well, when using the Power Saving Mode (PSM). The existing trade-off between the range and latency varies across different technologies (cf. Figure 2), aiming to cover a variety of use cases. This paper explores the aforementioned trade-offs and the conditions that enable the use of particular Internet of Things (IoT) wireless technologies in heterogeneous sensor-actuator networks for mid-range communication able to cover an industrial site ranging up to more than one kilometer in diameter.

Wireless Sensor Networks (WSNs) have been evaluated from different perspectives in the state of the art literature. However, significantly smaller amount of research is conducted in the context of IWSANs that have much more strict application requirements. An overview of key issues and challenges of wireless technologies in industrial networks is surveyed in References [1,12,13,14,15,16,17,18,19]. Communication requirements and a general profile of a wireless fieldbus for low level short-range factory automation systems are discussed in Reference [20]. References [13,21] discuss security and Quality of Service (QoS) perspectives of IWSAN in industrial automation. Furthermore, an in-depth review of recent advances in real-time IWSANs for industrial control systems is given in Reference [22], with a focus on WirelessHART. Reference [22] reviews real-time scheduling and analytic techniques for achieving real-time performance in Reference IWSANs. An extensive survey on wireless network design for control systems is presented in Reference [17], briefly reviewing a few of the existing wireless technologies in that context but mostly focusing on the joint design considerations of both control systems and wireless networks. A comparative examination of ZigBee, WirelessHART, ISA100.11a and WIA-PA in terms of network architecture and protocol design in the context of iwsan is given in Reference [19]. State of the art in Low-PowerWide-Area Network (LPWAN) solutions for Industrial Internet of Things (IIoT) services is explored in Reference [23].

This paper takes a different approach and interprets the wireless standards from the practical standpoint, offering the readers concrete values on achievable sampling rates, energy consumption, scalability and coverage in practice. Both standard specifications and product datasheets provide extensive low level data, and are insufficient on their own without additional empirical research. This paper quantifies the existing trade-offs in wireless technologies for wireless sensor and actuator networks with coverage of at least a couple of hundred meters, able to cover a production site or at least a large part of it. Range is crucial in process automation for all slave nodes to be able to reach a master node, considering that control is typically done by one or few master nodes (controllers) and a large number of slave nodes (sensors and actuators) that take part in bidirectional communication with the controller and are spatially distributed over the entire site. This paper presents cost, scalability, latency, reliability, range and energy consumption evaluation of wireless technologies with promising range and latency potential, including LoRa, IEEE 802.11ah (Wi-Fi HaLow), Narrowband-IoT (NB-IoT), WirelessHART, ISA100.11a, Bluetooth Low Energy (BLE) and 802.15.4g physical layer with 802.15.4e TSCH on data-link layer. These technologies offer the possibility of a dense heterogeneous wireless network deployment able to serve both actuators and sensors in critical applications, as well as provide an infrastructure for supervisory traffic. Along with the practical limitations of each technology with respect to the existing trade-offs between latency, throughput, coverage and scalability, a direct projection of the aforementioned wireless technologies to their key performance indicators is made, aiming to enable adequate network design in particular industrial applications.

The remainder of this article is organized as follows. The requirements and challenges that industrial networks must comply with are summarized in Section 2. Section 3 presents a general discussion of the key trade-offs in wireless network design in the context of the requirements, while in Section 4 the discussed trade-offs are quantified for each particular technology. Overall discussion and the experimental evaluation of energy consumption is presented in Section 5. Finally, Section 6 presents the conclusions.

## 2. Requirements and Challenges

The International Society of Automation (ISA) classified industrial systems into six classes [13] on the basis of data urgency and operational requirements. These classes range from critical control systems to monitoring systems, from the strictest requirements to the most relaxed ones respectively:

1. Safety systems—require immediate actions on events (usually in the order of tens or hundreds of μs or a few ms).
2. Closed loop regulatory systems - control the system via feedback loops operating either periodically or based on events. They may or may not have stricter timing requirements than safety systems.
3. Closed loop supervisory systems—similar to regulatory systems with the difference that the feedbacks are usually non-critical and event-based, for example, collecting statistical data and reacting only when a certain trend is observed by issuing a notification or alarm.
4. Open loop control systems—where sensors collect data and store it to the central database. An operator (human) analyzes the data and acts upon it if needed.
5. Alerting systems—send periodical or event-based alerts indicating different stages, for example, heating up the boiler and alerting every once in a while to indicate the progress.
6. Information gathering systems—collect the data (logging) and forward the logs to a server. These systems have no immediate operational consequence.

**Figure 3:**
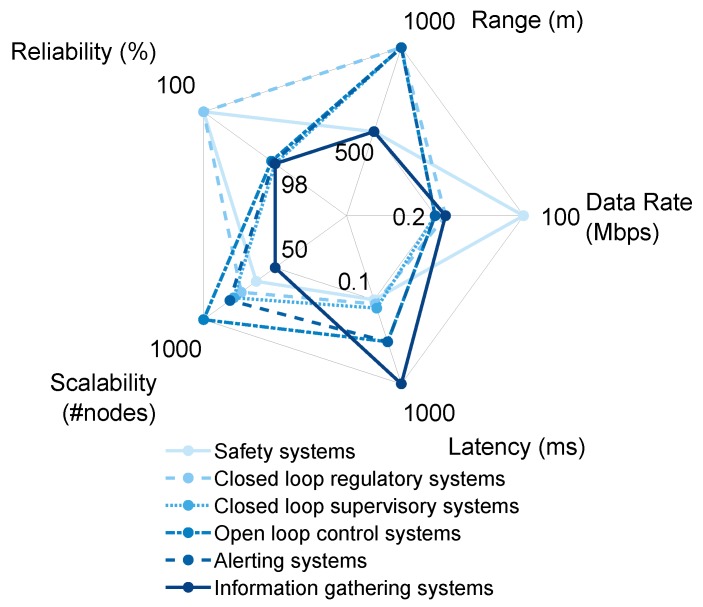
Different classes of industrial systems have significantly different performance requirements.

Wireless coverage of the entire industrial site may benefit classes 2–6, whereas class 1 requires a solution combining both ultra-high reliability, redundancy and ultra-low latency, which is infeasible with long range wireless considering the trade-offs. Performance requirements of different classes are depicted in Figure 3. For site-wide coverage, a range of at least a few hundred meters is needed. Site-wide coverage would enable multicasting measurements to several destinations, for example, actuators, supervision systems, databases, enabling the support of different services for several classes of industrial systems, making the network heterogeneous. Thus, site-wide IWSANs need to be scalable enough to accommodate new nodes and provide QoS, considering that they are expected to run for several decades. Different applications require different performance and services, as examples in Table 1 show.

Besides the key performance requirements illustrated in Figure 3, deployment cost, energy consumption, interoperability, QoS and service differentiation come to focus especially when considering heterogeneous networks. A wired fieldbus network is very expensive to deploy because of tens of kilometers of cables needed to connect devices to their master nodes, the time needed for deployment and the maintenance of such deployment. Lower deployment and implementation costs are the prime motivations for the transition from wired to wireless solutions wherever possible. Among wireless solutions, subscription fees for operator based networks also vary. Operator based solutions are generally not ideal for industrial purposes as the dependence on the operator in case of failure increases the repair time. However, reliable full-duplex operation of wired industrial networks is a large advantage over wireless technologies that are the subject of this article. Namely, reliability inherently suffers in the full-duplex wireless solutions because of the self-interference and increased interference from the neighbours [27]. Opting for half-duplex instead causes the inability to send and receive at the same time on the same channel, which in turn largely increases the latency in wireless networks. IWSANs must operate in real time to serve class two systems. Specifically, closed loop regulatory systems require IWSANs to sample, process and exchange the data between a sensor and an actuator in a time frame that is less than the cycle time of the loop, with typical values ranging from microseconds to hundreds of milliseconds (depending on the concrete process being controlled). Critical applications (classes 1 and 2) also require redundancy, resistance to noise and robustness against failure as they must ensure timely and successful delivery. In addition, the failure of one or a few nodes must not compromise the operation of the network as whole.

Many of the stated requirements are interconnected and there is no single technology that covers all of them simultaneously. The inevitable trade-offs, their causes and consequences are elaborated in the next section.

## 3. Trade-offs in Wireless Network Design

**Figure 4:**
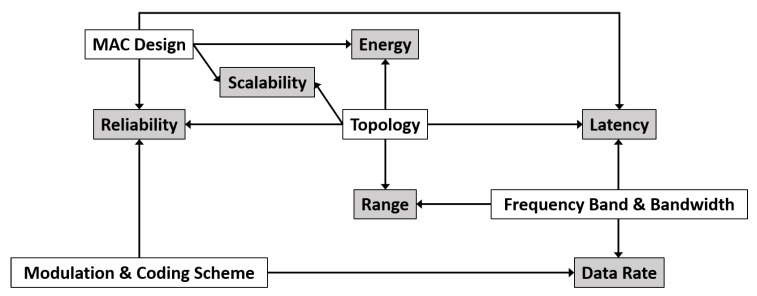
Network design choices (white rectangles) influence several performance properties simultaneously (grey rectangles), thus creating the trade-offs between them.

Providing wireless communication to heterogeneous applications, including the time-critical ones, over a wide industrial site is a conflicting task. For example, l All three are partly determined by the choice of frequency band and bandwidth but also with other design choices that create additional interlocks between the performance parameters. These trade-offs, illustrated in Figure 4, complicate the design of wireless solutions.

### 3.1. The Transmission Range

The transmission range is mainly determined by the transmission power, typically limited by regulations [11], the radio and propagation properties, as well as coding and modulation complexity. If a radio transmits at a constant power, lowering this complexity rate permits the correct decoding of a weaker or more distorted signal by a receiver, thus extending the transmission range. Also, higher frequency bands with more bandwidth available enable higher data rates and faster data transmission but they also have worse penetration capabilities which reduces the range in an industrial site full of obstacles. Range is largely determined by topology as well. Multi-hop topologies extend the range at the expense of latency, design complexity and energy consumption because of the need for synchronization of nodes, routing and so forth. In conclusion, low data rates at low frequencies and multi-hop topologies are prolonging the range but they all increase latency.

### 3.2. Latency

Latency is reduced by increasing the data rate, in turn enabled by more complex codings and larger bandwidths. Furthermore, multi-hop topologies increase the latency considering that forwarding and routing introduce additional delays. In addition, computing a new route when a link fails also introduces delay which can render multi-hop topologies useless in low-latency time-critical applications. Medium Access Control (MAC) design has a significant impact on latency as well, especially in IoT technologies where devices aim to sleep as long as possible to save energy, therefore delaying transmissions and receptions. MAC protocols can be classified into four classes: (1) Fixed Assignment Protocols where resources are divided among the nodes for a defined time duration, (2) Demand Assignment Protocols where resources are provided to a node on demand, (3) Random Access Protocols where resources are divided randomly and (4) Hybrid Protocols that combine fixed or demand assignment with random access. Fixed Assignment Protocols such as Time Division Multiple Access (TDMA) introduce determinism and achieve lower latency than random access protocols under very high load, but under low load they waste resources by inefficient usage of the channel time, where random access protocols achieve lower latency. Demand-based protocols are not suitable for low-latency time-critical communications given that explicitly asking for resources every time takes up bandwidth and adds up to latency. For heterogeneous industry applications, hybrid approaches are the most promising given that they aim to combine the benefits of both fixed assignment and random access protocols, while surpassing their limits at the same time and adapting to the network conditions. In addition, retransmissions need to be kept a minimum as they also increase the latency.

### 3.3. Reliability

Reliability is determined by topology, MAC design and Modulation and Coding Scheme (MCS). One of the major setbacks of wireless technologies in terms of reliability, in comparison to their wired counterparts, is the inter- and intra-technology interference on air which can cause collisions and increase packet loss. Technologies that operate in licensed bands reserve a part of the spectrum for themselves, mitigating the issue. However, spectrum is a scarce resource and it comes at a high price. Private deployments are not possible in reserved spectrum, disabling the possibility of local control over a network. Shared spectrum, on the other hand, can be shared by any number of technologies which can try and mitigate interference by channel hopping or using some MAC layer mechanisms such as Listen Before Talk (LBT). Furthermore, in a single-hop networks, the success probability is entirely dependent on a single link, opposed to multiple links in multi-hop networks. Reliability can be improved by employing both retransmissions and repetitions at the MAC layer, which also add to the latency. To reduce the number of retransmissions, error control techniques such as Forward Error Correction (FEC) can be used. Coding schemes and modulation largely define reliability. Coding rates create extra error checking bits that make modulation more reliable. Modulation schemes are more reliable as they have fewer points on the constellation diagram but also slower. That makes Binary Phase Shift Keying (BPSK) the slowest and the most reliable modulation compared to Quadrature Amplitude Modulation (QAM) and Quadrature Phase Shift Keying (QPSK), given that it only accommodates two points (one bit per burst). QPSK uses four constellations, whereas QAM can have any number of points. Any increase in the number of points on the constellation diagram reduces the space between them, leaving fewer margins for error. This makes QAM the fastest modulation scheme but more unreliable over longer distances.

### 3.4. Data Rate

Data rate is directly correlated with the available bandwidth and thus frequency band, on one hand, and with modulation and coding scheme on the other. More bandwidth enables higher data rates, while modulation techniques and coding schemes can further contribute to the achievable data rate by encoding more data into the signal. Unlicensed wireless technologies operate either in sub-GHz frequency bands (400 MHz, 800–900 MHz), in 2.4 GHz or in 5 GHz. Sub-GHz technologies generally (although not universally) use narrower channels (few hundred kHZ) than those in GHz frequency bands (22 MHz Wi-Fi, 2 MHz 802.15.4) and thus have more limited data rates than the GHz ones.

### 3.5. Energy Consumption

Energy consumption depends on data rate, topology and MAC design, as well as the hardware design of course. Low data rates result in long transmission times, which increases the energy consumption of the node and reduces the battery lifetime. Topology wise, nodes in multi-hop networks consume more energy than in single-hop networks given that, besides their own transmissions and receptions, they also need to forward other nodes’ packets. Energy-efficiency of data forwarding paths give the routing protocols a strong influence over energy consumption as well. Complex coding and decoding operations also contribute to energy consumption. For example, FEC has been omitted in commercial 802.15.4 based networks due to the energy consumption of the decoding operation. Nevertheless, employing FEC could reduce the overall energy consumption as less energy would be spent on retransmissions and rescheduling [28]. Besides, MAC design has a significant impact on energy consumption as it defines scheduling and hence the radio on and off times.

### 3.6. Scalability

Scalability is primarily determined by MAC design. Scheduling, contention resolution and other MAC mechanisms work together to provide maximum network capacity. In single-hop networks, the network capacity upon reaching the upper limit can only be extended by deploying more base stations. However, in practice the density of such base stations is limited. Multi-hop networks address this issue by allowing for wireless data forwarding, at the expense of overall throughput. In TDMA-based protocols, network density is limited by the need for synchronization and time division in combination with QoS requirements.

### 3.7. Spectrum Regulations

Another tackling design choice is the one between unlicensed Industrial, Scientific and Medical (ISM) and licensed bands. On the one hand, worldwide permitted unlicensed operation reduces the runtime costs but has no regulatory protection against interference by other wireless networks operating in the same frequency band. On the other hand, even though licensed bands prevent interference, they typically depend on an external operator. Therefore, network issues cannot be immediately resolved on site, the external operator needs to resolve them. This introduces administrative delays which are unaffordable in time-critical industrial applications. Communication technologies operating in the unlicensed spectrum are maintained and managed locally. However, several co-located or overlapping wireless networks operating in the same frequency band will interfere with each other and can experience decreased QoS and extensive packet loss [14]. In an effort to alleviate coexistence issues in unlicensed spectrum, regulatory bodies have issued a number of norms such as a Clear Channel Assessment (CCA) check before each transmission by all devices, that is, a device has to sense if the channel is free by energy detection or other types of Detect And Avoid (DAA) mechanisms [14]. Although the DAA mechanisms improve the coexistence between the contending wireless nodes and networks, collisions can still occur. Apart from collisions, medium sensing adds to the latency and introduces non-determinism due to the medium congestion. Aforementioned facts significantly limit the use of wireless solutions in closed loop control applications in automation industry. A limiting regulation is present in unlicensed sub-GHz spectrum as well. Devices with an operating range of 863–868 MHz in Europe, 916.5–927.5 MHz in Japan and 902–928 MHz in the US must comply with the maximum duty cycle limit of 2.8% and 10% for the, Access Point (AP) provided that they support LBT and Adaptive Frequency Agility (AFA) features, 1% otherwise [11].

## 4. Wireless Technologies for Industrial Applications

To support cyclic communication between sensors, actuators and controllers, sufficient throughput, latency and range is needed. This paper only considers wireless technologies that have the potential to enable real-time cyclic communication over a range comparable to the size of an industrial site, thus larger than a hundred meters. In line with that, we consider the promising IIoT technologies to be LoRa, IEEE 802.11ah and NB-IoT out of single-hop long range networks, WirelessHART, ISA100.11a, BLE and 802.15.4g/e physical (PHY) with 802.15.4e TSCH MAC out of long range multi-hop networks. The performance of each individual technology in terms of requirements presented in Section 2 and trade-offs presented in Section 3 is discussed below.

### 4.1. Long Range Networks

Single-hop long range networks that have the potential to enable real-time cyclic communication over a range comparable to the size of an industrial site are introduced in the remainder of this section.

#### 4.1.1. LoRa

LoRa is a proprietary wireless data communication technology which specifies a PHY layer only. A popular MAC for use with LoRa is the open LoRaWAN specification. LoRa PHY uses Semtech’s proprietary Chirp Spread Spectrum (CSS) radio modulation to reduce receiver complexity while achieving long range. CSS is resistant to Doppler effects and multipath fading. A LoRa receiver can decode transmissions ~20 dB below the noise floor, enabling very long communication distances while using very limited power. CSS is a spread spectrum technique where the signal is modulated by chirp pulses whose frequency linearly varies, parametrized by the orthogonal Spreading Factors (SFs), which can take values 7–12. The higher the SF, the longer packet transmission time and the more reliable its reception. Therefore, high SFs improve robustness against interference and counteract heavy multipath fading characteristic for indoor propagation and urban environments. This comes at the cost of low data rates and much higher energy consumption. Considering 125 kHz channels, the data rates range from 0.25 kbps to 5.47 kbps. These very low rates result in long transmission times (and medium usage) even for small packets. For example, a 17-byte sensor reading would take over 1.5 s to transmit at SF12 and cca. 70 ms at SF7. Combining the LoRaWAN MAC and LoRa PHY data rates results in an rather low network capacity per gateway of less than 0.02 MB per hour [29].

An experimental study on the range of LoRaWAN [30] showed that it can achieve ranges up to 7.5 km using sf10 and packets with 10 bytes of payload, resulting in 0% Packet Error Rate (PER) and −126 dBm Received Signal Strength Indication (RSSI). When using the specification of the Wireless M-Bus according to EM13757-4, 50 bytes of payload, Frequency Shift Keying (FSK) modulation with an FSK deviation value of 50 kHz and a data rate of 100 kbps, LoRa achieves up to 1.35 km of range with 0% PER and up to 3.6 km of range for <10% PER.

LoRaWAN is based on pure ALOHA, thus collisions pose the biggest issue in such networks given the long air-times. According to LoRaWAN, edge-devices’ downloading needs determine their class:

- Class A devices have a single ReceiveWindow (RW) scheduled immediately after a corresponding uplink connection,
- Class B devices can schedule additional RWs,
- Class C devices continuously listen and can receive almost anytime.

The more RWs, the more energy devices consume, that is, the power consumption increases over the classes A through C.

LoRaWAN supports both confirmed and unconfirmed messages. However, downlink capability of LoRaWAN networks is highly limited. With an increasing traffic load, RWs for sending acknowledgements (ACKs) to confirmed messages are more frequently missed as the gateway cannot transmit at the start of a RW due to the duty cycle restrictions. When a gateway sends an ACK in either RW1 or RW2, it aborts all ongoing receptions further decreasing the Packet Delivery Ratio (PDR) [31]. Currently, various scheduling solutions are being investigated that aim to improve LoRaWAN capacity and reliability, properties that are of interest to industrial IWSANs [32]. At this moment, even with the adaptation of LoRa/LoRaWAN to TSCH mechanism, this technology can only fulfill requirements for those industrial application scenarios classified to classes 3–6 that need cycle time in the order of seconds in the best case scenario, or even minutes for very dense deployments [33]. Reference [34] analyzed end-to-end latency of LoRaWAN in IIoT use cases and showed that LoRaWAN may be applicable for a subset of IIoT use cases where lower SFs can achieve end-to-end delay below 400 ms. Long distance transmissions ranging up to 15 km require a high SF and result in the end-to-end latency well above one second, which is far beyond real-time availability of sensor data.

In conclusion, the LoRa PHY allows long range, but the low network capacity and current class A MAC design restricts use cases to those with a combination of low data requirements, a majority of uplink traffic and overall traffic volumes that remain far below the theoretical capacity.

#### 4.1.2. IEEE 802.11ah/Wi-Fi HaLow

IEEE 802.11ah, marketed as Wi-Fi HaLow, can serve up to 8192 stations per AP, a much higher value than the previous 802.11 iterations. In addition to the 1 km coverage, its relay functionality can further increase both the network size and coverage. To limit interference between stations and collisions, it introduces the Restricted Access Window (RAW) mechanism which combines the deterministic and the stochastic channel access. RAW restricts the channel access for a specified group of stations assigned to the time slots within the RAW. Stations assigned to specific slots within a RAW contend for the medium in their corresponding slots employing Enhanced Distributed Channel Access (EDCA)/Distributed Coordination Function (DCF). The stations are not allowed to contend for the medium in RAW slots they are not assigned to. RAW configuration and assignment is configurable and can change every beacon interval. RAW can reserve channel time for any group of stations. It improves throughput in dense IoT networks where many stations contend for the medium simultaneously. RAWs can contribute to introducing determinism in a network, if configured accordingly. However, an arbitrary RAW configuration can also degrade network performance if RAW is not configured with respect to the traffic patterns of the end devices. RAW duration can be configured to any value between 500 μs and the beacon interval.

IEEE 802.11ah demonstrates robustness in industrial environments given that it has better penetration capabilities than 2.4 GHz technologies due to sub-GHz frequency bands. It employs Wi-Fi WPA3 for security and can be used for over-the-air software updates [35], besides monitoring and control.

The minimal feasible cycle time in a 802.11ah network equals 2 or 4 beacon intervals, given that 2 to 4 hops are needed to complete a single cycle in a control loop in a star network: two if the controller is wired to the AP and four otherwise. Each hop can only be executed in a single beacon interval. Beacon intervals can be reduced in order to support low-latency loops but reducing the beacon intervals makes the spectrum usage less efficient [36]. The minimal cycle time for a single 99.99% reliable control loop with a wired controller is 32 ms and it can operate alongside 75 sensors reporting 64-byte measurements every 1 s. Longer cycles enable the reliable support of more control loops, for example, 4 control loops with 64 ms cycle time and 70 sensors reporting every 1 s. IEEE 802.11ah can offer differentiated QoS to different types of end nodes in a dense deployments [37,38]. In addition to classes 3–6, this technology may be capable of providing QoS to class 2 as well, supporting low latency closed loops (<100 ms) along with other non-critical traffic. However, the lower the latency requirement, the fewer nodes the network can support due to the used up bandwidth. Hence, there is a trade-off between the scalability and the latency that can be supported in practice. Given the lack of available hardware at the market at this time, it is yet to be seen how does this technology perform in the real world, outside of simulation studies.

#### 4.1.3. Narrowband-IoT

NB-IoT aims to offer deployment flexibility allowing an operator to use only a small part of the available spectrum for the technology. It targets ultra-low-end IoT applications. NB-IoT supports three coverage classes, namely (1) normal, (2) robust and (3) extreme coverage class. Those correspond to the Minimum Coupling Losses (MCLs) of 144 dB, 158 dB and 164 dB respectively. In NB-IoT, the uplink latency consists of the system synchronization, broadcast information reading, random access, resource allocation, data transmission and feedback response [39]. In uplink, NB-IoT makes use of single- (20 kbps) and multi-tone (250 kbps) channels. A single-tone technology implies either 12 or 48 continuous sub-carriers with sub-carrier spacing of either 15 kHz or 3.75 kHz, respectively. Multi-tone technology implies 12 continuous sub-carriers, with 15 kHz spacing, grouped in 3, 6 or 12 continuous sub-carriers. Spacing of 3.75 kHz results in higher coverage than 15 kHz spacing due to the higher power spectral density, which makes the cell capacity 8% larger for 3.75 kHz-spacing [40]. In downlink, Orthogonal Frequency-Division Multiple-Access (OFDMA) is employed with sub-carrier spacing of 15 kHz.

Recent measurements on an actual public NB-IoT network showed that the achievable application layer throughput is significantly lower, at around 10 and 15 kbps for uplink and downlink, respectively [41]. In terms of latency, a one-way latency of about 50 ms could be achieved under perfect channel conditions for small packets of 8 bytes. However, for deep indoor scenarios, latency increased to around 16 s for uplink and 8 s for downlink transmissions. Another experimental evaluation of NB-IoT using two different commercial platforms in a public NB-IoT network has observed the inconsistency in performance metrics, namely the energy consumption and latency [42]. The variability of energy consumption results in an imprecise predictability of battery life and causes a difference in performance of similar devices. Although NB-IoT is designed for delay-insensitive applications, some cases cannot tolerate the variability of latency amounting to tens of seconds or even minutes. Guaranteed reliability in NB-IoT comes at a cost of variability [42].

NB-IoT is superior than most of the competition in terms of range, security and availability. However, its unpredictable latencies that can be in the order of seconds make it applicable for latency insensitive processes only. Its random resource reservation procedures make the connection latency high in dense networks. When connected, its throughput and latency can significantly vary depending on channel conditions due to the repetitions and changes in MCSes. Even though the hardware is inexpensive, NB-IoT comes with a subscription fee, while all other technologies listed in Table 2 and Table 3 can be deployed privately. In conclusion, it is significantly more robust than competition which makes it suitable for class 2 latency insensitive applications. It can also be used in classes 3–6.

### 4.2. Long Range Multi-hop Networks

Multi-hop networks not only extend the range but also substantially contribute to the reliability as in mesh networks there are (typically) 2 or more paths from the source to the destination, meaning that the loss of a link will not result in a communication failure like in single-hop networks. However, nodes in multi-hop networks consume more energy because aside from their own packets, they also must transmit and receive (forward) other nodes’ packets. Routing the packets adds up to latency and complexity. Thus, even though the scalability of multi-hop networks is not explicitly limited, deployments typically do not include more than 5 hops. The theoretical limit on the maximal network density is based on the addressing space, which for most of the analyzed technologies is $2^{32}$, thus considered unlimited. However, the practical capacity limit is determined by the network latency and individual device’s energy consumption. They both increase with the number of the communication links in the network. In addition, bottlenecks can occur at one or more devices that communicate directly with the gateway in a mesh network. Because of this, data traffic can significantly increase resulting in the increase of the devices’ energy consumption. Also, the maximum achievable update rate is greatly influenced by the network density given that high update rates generate more traffic than low update rates.

#### 4.2.1. WirelessHART

Along with the PHY, MAC, network and transport layer, the WirelessHART protocol stack also includes the application layer. The application layer is HART, which is compatible with existing wired HART solutions.

Both WirelessHART and ISA100.11a combine TDMA and frequency hopping at the MAC layer. To access the channel, WirelessHART uses a two-dimensional matrix consisting of time slots and 15 available channels. Time slots are grouped in superframes which periodically repeat throughout the entire network lifetime. Variable-length superframes are also supported and at least one superframe must be enabled. Superframes can be added or removed during the operation of the network and they are managed by the network/system manager. Time slot duration is fixed to 10 ms in WirelessHART. This scheme allows multiple devices to transmit data at the same time using different channels. However, a single device can only make use of a single channel per time slot. WirelessHART’s (single) time slotted channel hopping mechanism also identifies noisy channels and blacklists them, thereby greatly improving the network reliability. In addition, this technology can achieve up to 99.999% reliability by supporting mesh topologies [43]. Security in WirelessHART communication is provided by the 128-bit Advanced Encryption Standard (AES) authentication.

WirelessHART is the oldest and the most experimentally evaluated wireless solution for IIoT [44,45,46,47]. A study has demonstrated control over WirelessHART with the update rate of 20 ms [45]. However, this and similar studies evaluate the simplest variant of a WirelessHART network comprising of a sensor, an actuator, an AP, a gateway (GW) and a network manager. Such a low update rate is achievable only in star topologies and with very short superframes that disable scaling the network up. Even though not more than 5 hops are advised in industrial networks, scaling WirelessHART is possible (1) using multiple APs and (2) using several WirelessHART gateways connected to a HART-over-IP backbone. This is very much needed in dense industrial environments as only 8 devices with 500 ms reporting period can be supported by a single AP [48]. In practice, a single WirelessHART AP can support 25, 50, 80 or 100 devices at 1 s, 2 s, 4 s and 8 s reporting period respectively. Both bidirectional (cyclic) traffic and an increment in hops doubles the latency or halves the number of nodes. Hence, WirelessHART is eligible for use in classes 2–6 of industrial systems, although it is limited either by latency or by scale.

#### 4.2.2. ISA100.11a

The ISA100.11a protocol is designed for secure and reliable wireless operation for noncritical monitoring, alerting, supervisory control, open- and closed-loop control applications (classes 2–6). Although it is in many aspects similar to WirelessHART, unlike WirelessHART it provides flexibility for customizing the operation of a system. It is based on 802.15.4 PHY and achieves a single-hop range up to 100 m. The ISA100.11a utilizes TDMA and Carrier Sense Multiple Access with Collision Avoidance (CSMA/CA) on MAC layer, as well as graph routing and channel hopping which improves reliability by avoiding busy channels. The CSMA/CA mode is commonly used for retries, association requests, exception reporting and burst traffic. The use of any single channel is reduced by the time-synchronized slots and channel hopping, resulting in the improvement of ISA100.11a’s coexistence with other networks in the shared spectrum. Communication takes place in time-slots with configurable constant lengths, varying from 10 ms to 12 ms. Time-slots are grouped in a superframe that periodically repeats in time. The length of the superframe is configurable and can differ for each node. Generally, long-period superframes result in high latency and low bandwidth. However, they conserve energy and contribute to the outspread allocation of digital bandwidth. Three methods are employed for limiting the use of undesirable radio channels and reducing the interference with other wireless networks: (1) CCA, (2) spectrum management and (3) and adaptive channel hopping. There are 15 (16 if the optional channel 26 is enabled) available channels to chose from in each time-slot. Three channel hopping sequences are defined, along with five hopping patterns.

In dense deployments, ISA100.11a devices can join the network faster than WirelessHART devices, as shown in Reference [49]. The WirelessHART network manager allows the initiation of the join process for each transmission opportunity (TXOP) and allocates the resources for publishing data during the join process. On the other hand, ISA100.11a employs dedicated advertisement links for join process initiation. At the network startup, the ISA100.11a gateway acts as a join proxy. However, each joined device may act as a join proxy, thus allowing more join opportunities. This results in faster joining of ISA100.11a devices in a dense deployment but not when there are only few devices. Reference [49] observed ISA100.11a to be slightly more reliable than WirelessHART, but also to have larger latency than WirelessHART in a cyclic communication.

Unlike WirelessHART, not all devices in ISA100.11a network must have routing capability. Without it, devices must be within one hop of a routing-capable device or the gateway. This significantly constrains the network’s re-routing capability in case of link fading due to changes or movements in the plant, making the network less adaptable to changes. In addition, even though the standard does not impose a limit on the number of nodes, in practice it is only possible to connect up to 10 devices reporting every 0.5 s to a single AP, 25 devices reporting every 1 s, 50 at 2 s, 80 at 4 s or 100 at 10 s [48]. Both bidirectional (cyclic) traffic and an increment in hops doubles the latency or halves the number of nodes. This makes ISA100.11a suitable for application to classes 3–6, as well as to class 2 assuming the specific requirements of a class 2 application are in line with the latency and scale constrains of this technology.

#### 4.2.3. Bluetooth Low Energy

Unlike classic Bluetooth which was designed as a point to point wire replacement, BLE provides increased range with 1 Mbps data rates and recently introduced meshing. An experimental study [50] observed the range of around 50 m indoors. The observed outdoors range for non line of sight scenarios was 123 m and 165 m for 0 dBm and 9 dBm transmission gain respectively, versus 490 m and 790 m in line of sight scenarios. Instead of Bluetooth’s 1-MHz wide 79 channels, BLE uses 40 channels of 2 MHz. It employs frequency hopping over 37 channels for (bidirectional) communication and 3 for (unidirectional) advertising. Two MAC layer roles are defined for the devices in BLE, master and slave. The master coordinates the medium access using a TDMA-based polling mechanism, in which it periodically polls the slaves in their corresponding *Connection Events (CEs)* which are uniformly spaced within a configurable periodic interval called a *Connection Interval (CI)*. Frequency hopping is employed in a way that each ce uses a different channel. The standard defines the CI duration as a multiple of 1.25 ms in the range from 7.5 ms to 4 s. Therefore, the minimal cycle time equals the CI in a BLE control loop. Considering that the sample/update rate in an industrial system should be 3–4 times faster than the process time constant for condition monitoring and open loop control and 4–10 times faster for regulatory closed loop control, the minimal cycle times achievable with BLE increase to 22.5 ms–30 ms for non-critical traffic and 30 ms–75 ms for regulatory loops. BLE is theoretically capable of supporting 6 control loops exchanging 47-byte (max. size) packets with the minimal CI. Extending the CI for *n* time units of 1.25 ms results in *S* control loops, as follows:

(1) $$S=\lfloor \frac{7.5\;\mathrm{ms}+n\cdot 1.25\;\mathrm{ms}+T_{GB}}{2\cdot 0.376\;\mathrm{ms}+0.15\;\mathrm{ms}+T_{GB}}\rfloor$$

where 0.376 ms is the transmission duration of a 47-byte packet at 1 Mbps, 0.15 ms stands for an Inter-Frame Spacing (IFS) and $T_{GB}$ (in ms) for implementation-specific guard band between two successive CEs. This reasoning is based on the one-to-one mapping between control loops and CEs in a single CI. Besides 2S transmissions in a CI, we must fit *S* IFSs between successive transmissions in CEs and S−1 guard bands between CEs as well.

Bluetooth 5 provides the options to double the speed to 2 Mbit/s at the expense of range or up to fourfold the range at the expense of data rate and eightfold the data broadcasting capacity of transmissions by increasing the packet lengths. However, BLE’s limited range makes it unsuitable for large scale IIoT deployments, as over 1000 basestations would have to be deployed to cover the same area as a single Wi-Fi HaLow basestation. To overcome this issue, Bluetooth introduced meshing. Communication in the Bluetooth Mesh standard in implemented using BLE advertising and scanning on the 3 advertisement channels. The standard uses a flooding mechanism where each node in the network repeats incoming messages, relaying them until the destination. Unlike in normal BLE advertising, Bluetooth Mesh transmissions are not scheduled based on an advertising interval. Instead, the nodes transmit after a random backoff time. In a Bluetooth Mesh network, increasing the number of relaying neighbors from 1 to 10 reduces the 2-hop round-trip time (RTT) from 47 ms to 33 ms for 41-byte packets and 10 ms scanning interval [51]. Increasing the number of hops from 1 to 4 increases the RTT from 22 ms 88 ms. This is because the latency is influenced the most by the random backoff mechanism. However, the absence of a random backoff would increase the collision probability, decreasing the reliability and scalability of Bluetooth Mesh networks which makes it suitable only for classes 4–6 of industrial applications.

#### 4.2.4. Time Slotted Channel Hopping

A MAC layer with TSCH can operate on top of both 2.4 GHz- and sub-GHz 802.15.4-based PHY. Assuming up to 4 hops, 802.15.4 networks that operate in unlicensed 2.4 GHz frequency bands can achieve a range of up to 200 m. On the other hand, their sub-GHz counterparts based on 802.15.4 g can reach up to 1 km single-hop range [52].

In TSCH mode, time is slotted and time slots are grouped in slot frames. Both the time-slot duration and the number of slots in the slot frame are configurable. Slot duration is determined by the time needed to transmit a packet and receive an ack and typically amounts 6 ms–10 ms. The length of the slot frame is based on the trade-off between energy consumption and throughput. Decreasing the slot frames increases both the energy consumption and the throughput and vice versa. For cyclic communication in a closed loop, a node would need two slots in a slot frame. The cycle could repeat every slot frame, thus the cycle time trades off with the number of nodes in the network. A network of two nodes could exchange packets with ~20 ms cycle time but 25 closed loops would increase the minimal cycle time to 500 ms. Considering the best practices regarding the sampling in industrial communications, the minimal cycle times achievable with TSCH in practice increase to 60 ms–80 ms for non-critical traffic and 80 ms–200 ms for a single regulatory loop (2 s–5 s for 25 loops).

Besides time slotting, TSCH also utilizes channel hopping to diminish the impact of interference and channel fading. Nodes communicate with each other over 16 orthogonal channels and retransmit packets on different channels if initial attempts fail, thereby increasing reliability. Channel hopping scheme could benefit the scalability and the aggregate throughput of the network if all channels were used in a same time slot. However, this benefit is largely limited in industrial networks given that all slave nodes communicate with the same master node in the network, as illustrated in Figure 1. Unless a master node is able to send and receive on multiple channels simultaneously, which is not the default capability, nodes will be forced not to transmit simultaneously, regardless of the channel hopping scheme.

Furthermore, reliability of 2.4 GHz TSCH networks may be significantly reduced in the presence of interference of co-located Wi-Fi networks, as shown in Reference [53]. When only a few channels in the TSCH matrix are affected by interference, only an increase of the transmission latency due to retransmissions could be expected. However in reality, the probability of eventual packet dropout is no longer negligible because of the quite low default retry limit (4) and the fact that one Wi-Fi channel overlaps many IEEE 802.15.4 channels (four to five). As Reference [53] shows, this results in an increase of packet losses, in turn reducing the reliability. Reliability could be increased by increasing the retry limit, hence trading off the latency and power consumption.

In conclusion, TSCH networks utilize the schedule on each node to achieve determinism and robustness against channel fading, as well as to conserve energy. That robustness and determinism makes TSCH suitable for class 2–6 industrial applications, assuming it can comply with the range requirement. However, there is a strong trade-off between latency and scalability, given that multiple channels cannot be used in parallel by different slave nodes due to the centralized master node.

## 5. Discussions

Key specifications of each technology introduced in Section 4 are listed in Table 2 and Table 3. Note that not all numbers in Table 2 and Table 3 can be taken for granted as they do not depict the trade-offs, but only present theoretical limits. NB-IoT operates in licensed spectrum. It can reach as far as its cellular infrastructure goes, and it is inherently more reliable than technologies that operate in shared spectrum. LoRa, IEEE 802.11ah and 802.15.4g operate in unlicensed sub-GHz frequency bands, which makes their long range a feature of their physical layers. LoRa operates in 863–870 MHz frequency band in Europe, offering three sub-bands at 864 MHz, 867 MHz and 868 MHz. Five 125 kHz channels are defined in 867 MHz band and three in 864 MHz and 868 MHz band [54,55]. The three default channels in 868 MHz band are to be implemented by every node, whereas the rest are optional. Wi-Fi HaLow operates in 863–868 MHz band in Europe and defines five 1-MHz channels and two 2-MHz channels [56]. Considering this, it is clear that Wi-Fi HaLow and LoRa’s optional channels overlap in 3 out of 7 Wi-Fi HaLow channels and do not interfere with each other otherwise, which makes their parallel deployments possible. However, Wi-Fi HaLow can severely interfere with 802.15.4g [57], given that they operate in the same bands. The 2.4 GHz unlicensed frequency band is widely utilized today and the technologies that operate in this band must pay special attention to coexistence. WirelessHART, ISA100.11a and 802.15.4e TSCH are all based on the 802.15.4 2.4 GHz PHY layer that operates in sixteen 2 MHz-wide and 5 MHz-spaced channels in 2.4 GHz frequency band. They all employ frequency-hopping to improve the reliability of their transmissions, as they make use of exactly the same spectrum. Given their shorter single-hop ranges, these technologies employ multi-hop topologies to extend their range.

Wireless networks have the benefit of mitigating cabling for communication but the question of power cables still remains. Ideally, power cables could also be mitigated when the devices can live long enough drawing the power from the batteries available today. This is feasible for sufficiently large cycle times. Therefore, it is important to also consider the energy consumption. To evaluate the possibility of entirely wireless deployments that do not need power cables, an experimental energy efficiency comparison between LoRa, NB-IoT, IEEE 802.11ah and IEEE 802.15.4g (Wi-SUN with TSCH) was performed. The experiments combine energy consumption values of state-of-the-art off-the-shelf radios obtained from their data sheet (cf. Table 4), with simulated performance analysis. At the time of this study, no off-the-shelf radio was available for Wi-Fi HaLow and the same radio was assumed as for 802.15.4g (Atmel AT86RF215), as it supports the required modulation and coding schemes. The simulation experiments were performed using the ns-3 network simulator for LoRa [58], NB-IoT [59] and Wi-Fi HaLow [60]. Using the 6TiSCH simulator, 802.15.4g was evaluated [61]. A single device and a single gateway/AP is considered in the simulations. Hence, the results do not take into account scalability or contention. To maintain simplicity, predictability and comparability of the results, the experiments did not take into account any packet loss due to propagation errors, interference or collisions. As such, the PHY and channel contribution to the energy consumption is limited to the time-on-air of the radio for the reported packet sizes (including preamble and PHY/MAC header). The main contribution of the results pertains to the energy consumption of the MAC layer protocols of the considered technologies. For this reason, we used a simplified linear battery discharging model.

**Figure 5:**
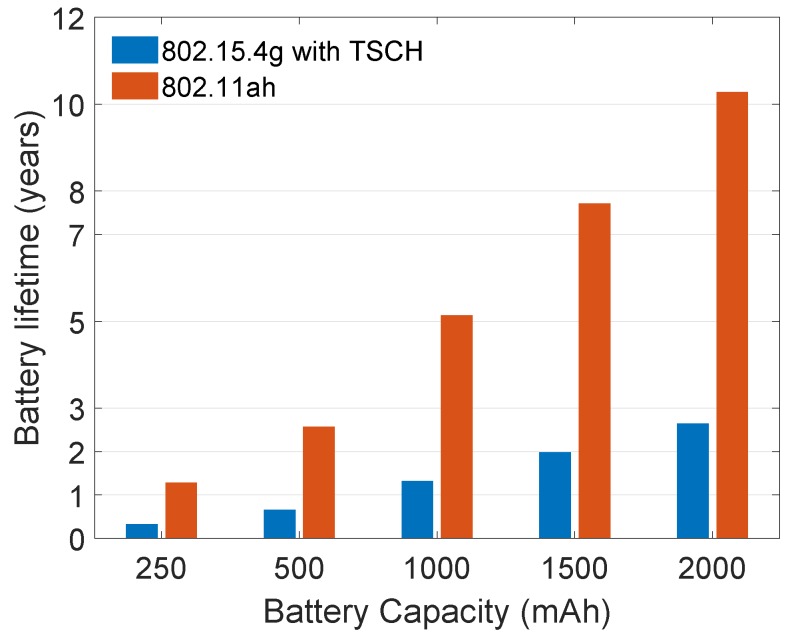
Battery lifetime of a device (both radio and microcontroller) transmitting once every 10 min for long-range technologies (>1 km).

**Figure 6:**
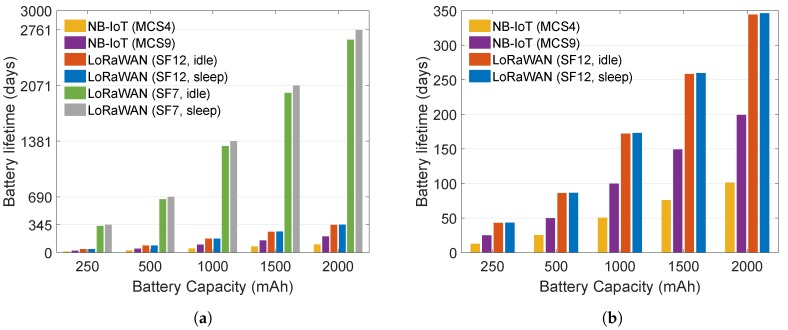
Battery lifetime of a device (both radio and microcontroller) transmitting once every 10 min for long-range technologies (<10 km). (**b**) is a subset of (**a**) where LoRaWAN SF7 is omitted for clarity.

Wi-Fi HaLow is significantly more energy efficient than 802.15.4g with TSCH. This improvement is due to the higher supported data rate that leads to shorter transmission times. This result is also reflected in the predicted battery lifetime shown in Figure 5. Given a sufficiently large battery of 2000 mAh, the lifetime of Wi-Fi HaLow is expected to be above 10 years for a 10-min transmission interval, without battery replacement. For 802.15.4g, battery life expectancy is under 3 years in the same scenario. For the long-range contenders, it is under a year, as shown in Figure 6b, except the LoRaWAN sf7 that can live up to 7.5 years (cf. Figure 6a). The compared configurations of 802.15.4g and 802.11ah do not represent the best case scenario regarding energy consumption, as both technologies are configured to use low data rates. However, the chosen settings result in similar coverage range. In a line-of-sight scenario, 802.15.4g FSK-50 reaches a good PDR up to 700 m, while FSK-200 goes up to 420 m [62]. For 802.11ah [63], mcs10 (150 kbps) has a range of 700 m to more than 1000 m. For a higher data rate, 802.15.4g will achieve better energy efficiency but it’s achievable range would be very different from that of mcs10 of 802.11ah. Similarly, 802.11ah will achieve better energy efficiency for higher data rates (mcs10 represents the lowest possible data rate and thus the worst case).

Figure 6 illustrates the benefits of low power modes in the design of a technology. As shown in the Figure, the lifetime of LoRaWAN devices is much higher than that of NB-IoT. Power consumption of LoRaWAN devices in transmission (TX)/reception (RX) mode is the dominant factor of the overall consumption, considering the very low power consumption in the idle/sleep mode (cf. Table 4). This results in a large difference in lifetime between LoRaWAN devices that use SF7 and SF12. The difference between SF7 and SF12 is especially large when the transmissions are frequent. The rarer the transmissions, the smaller the difference between SF7 and SF12 as idle/sleep time prevails and the TX/RX time becomes less significant in comparison to the idle/sleep time.

The larger difference in battery lifetime between NB-IoT mcs9 and mcs4 occurs due to the better efficiency of mcs9 which reflects not only in data TX/RX but also in signaling between the transmissions which adds up to the difference. The difference between mcs9 and mcs4 also reduces as the traffic interval increases, similarly as the difference between spreading factors of LoRaWAN. NB-IoT has higher data rate than LoRaWAN, thus it is more efficient in terms of TX/RX. However, the NB-IoT hardware is less efficient in terms of energy consumption in all four modes, so LoRaWAN becomes more energy efficient relative to NB-IoT. Higher data rates of NB-IoT cannot compensate for the energy efficiency of LoRaWAN in the evaluated scenarios.

It is important to note that the shelf life also affects the battery life. The lifetimes illustrated in Figure 5 and Figure 6 represent the ideal cases which only take into account the consumption of a radio and a microcontroller. Hence, the presented results do not take into account energy consumption of peripherals, self-discharge of battery nor other power drains. For example, when considering only radio and microcontroller, NB-IoT platform using mcs4 and mcs9 would need around 230 mAh and 155 mAh respectively to run for three years. For the same time period, LoRaWAN radio and microcontroller using SF7 and SF12 would consume 8 mAh and 47 mAh, when ignoring other power drains and employing sleep mode (45 mAh and 83 mAh with idle mode). However, the above mentioned factors need to be taken into account when estimating very long battery lives as they make the largest part of energy consumption over a long time.

This paper focused mostly on PHY and MAC layer features of the considered wireless technologies, even though some technologies also address commissioning, security, roaming and other higher layer features. In addition to PHY and MAC, LoRaWAN defines a complete network architecture, various device types, commissioning and security (network and application). Parameter configurations and network management are generally implementer specific for all the considered technologies. LoRaWAN is configurable in terms of many parameters such as:

- spreading factor (and thus data rate): fixed choice or adaptive,
- reliability: ACKs or multiple transmissions of the same packet without downlink ACKs,
- higher layer logic: raw payload or Internet Protocol (IP) compliant stack.

Choosing the network setup for a certain scenario is not always straightforward, which motivated using optimization techniques to choose the optimal parameters [64]. Moreover, LoRaWAN does not impose the coordination of transmissions of class A devices, which might impact scalability in real deployments. Similarly, Wi-Fi HaLow defines PHY and MAC layer, as well as layer 2 security upon connecting. On top, IP compliant stack is to be used. Wi-Fi HaLow is highly configurable, defining a multitude of different parameters to be configured such as MCS, RAW, Traffic Indication Map (TIM) and others [37,65,66]. The configurable parameters can significantly influence the performance and the lifetime of the network. Higher layers of NB-IoT can be both IP- and non-IP compliant. NB-IoT also defines some configurable parameters such as extended Discontinuous Reception (eDRX) and PSM timers. However, other settings are under control of the network operator. The standard IEEE 802.15.4g/e TSCH defines PHY and MAC, with TSCH MAC layer specifying how to execute the schedule but not how to define it. Given that a schedule significantly impacts the network performance, there are standardization efforts in Internet Engineering Task Force (IETF) on scheduling functions but there is also a choice between centralized and decentralized scheduling. WirelessHART and ISA100.11a share a similar concept as TSCH. They have self-configuration capabilities greatly simplifying the deployment. They define the full stack and are centrally managed by the network manager that makes use of commands defined by the standard for network management [67]. They are out there for a long while and are quite well understood. BLE also defines the entire stack as well as other aspects such as commissioning. BLE has configurable parameters such as advertisement interval and connection interval that influence its performance. Different combinations of the configurable parameters can result in a different performance of any technology in various scenarios, thus the interdependence of the configurable parameters needs to be empirically examined.

## 6. Conclusions

LoRa, IEEE 802.11ah, NB-IoT, BLE, WirelessHART, ISA100.11a and TSCH can provide site-wide network coverage if configured and deployed accordingly, at the cost of data rate and/or latency and/or scalability. The trade-offs of the evaluated technologies are basically equivalent qualitatively but greatly vary in quantity, as elaborated in Section 4. NB-IoT and LoRa have potential to serve non-critical class 4–6 systems. On top, NB-IoT is more suitable for frequent downlink traffic because of LoRaWAN’s downlink limitations, which makes it eligible for class 3 systems, as well as latency insensitive class class 2 systems (regulating very slow processes). However, NB-IoT typically comes with a subscription fee and due to the operator involvement, maintenance is slower. Thus, both have pros and cons, as well as similarities. BLE could serve supervising non-critical systems given its inherent unreliability in mesh topology and range limitations in star. TSCH, on the other hand, provides necessary reliability for critical services but its scale and latency are limited by the typical industrial fieldbus topology. WirelessHART, 802.15.4e TSCH and ISA100.11a are similar in terms of both reliability and latency. They all operate in the same frequency band and they all employ channel hopping to improve coexistence. Although they provide high data rates in comparison to other technologies, mesh topology increases the latency and limits the scalability. Still, these technologies are being deployed in class 2–6 systems. Finally, Wi-Fi HaLow trades data rate for range and latency but can still support the lowest latencies compared to reviewed technologies in networks of limited scale. Its star topology and novel MAC design contribute to high energy efficiency. Other technologies exhibit various trade-offs as well, ergo the choice of appropriate technology for a certain application is very challenging, given the number of options. This paper quantitatively evaluates the aforementioned technologies, outlining their main advantages and disadvantages and enabling the appropriate choice of the technology for an iwsan of interest, considering the specific latency, reliability, scalability, energy consumption, data rate and coverage requirements.

## Figures and Tables

**Table 1:** Cycle time and communication range requirements broadly vary over industrial automation use-cases [12,24,25,26].

Application	Range [m]	Cycle Time
Building automation	10–200	100 ms—seconds
Monitoring and supervision	100–1000	seconds—days
Process control	50–500	10–1000 ms
Factory automation	10–50	0.5–100 ms
Automotive	1–10	1–100 ms
Interlocking and control	50–100	10–250 ms
Power-system protection	100–10 k	0.01 μs–50 ms
Event-based control	10–100	1–100 ms

**Table 2:** Technical properties (top) and key performance indicators (bottom) of sub-GHz wireless technologies for the IIoT domain.

	LoRa	IEEE 802.11ah	NB-IoT	802.15.4g TSCH
**Band**	unlicensed sub-GHz	unlicensed sub-GHz	licensed (LTE band)	unlicensed sub-GHz
**Bandwidth**	125 kHz/250 kHz	1/2/4/8/16 MHz	180 kHz	200 kHz–1.25 MHz
**Topology**	star-of-stars	star/tree	cellular	star, p2p mesh
**Deployment**	private/operator-based	private	operator-based	private
**MAC**	LoRaWAN	hybrid	LTE based	TSCH
		EDCA/DCF	OFDMA (DL) & SC-FDMA (UL)	
**Retransmissions**	yes	yes	yes	yes
**Reliability mechanisms**	orthogonal SFs	FEC, WPA1 (MIC), WPA2 (CCM)	FEC, ARQ	FSK/O-QPSK/OFDM
	32-bit MIC	WPA3 (BIP-GMAC-256)		
**Range**	15 km	1 km	20 km	1 km
**Nodes per network**	unlimited	8192	52,247 per cell	6000
**Data rate**	250 bps–5.5 kbps/11 kbps/50 kbps	150 kbps–78 Mbps	<250 kbps	6.25 kbps–800 kbps
**Min. cycle time**	>1 s	>20 ms	>1.6 s	> 20 ms

Orthogonal Frequency-Division Multiplexing (OFDM); Orthogonal Frequency-Division Multiple Access (OFDMA); Single Carrier FDMA (SC-FDMA); Uplink (UL); Downlink (DL); Automatic Repeat reQuest (ARQ); Offset QPSK (OQPSK); Counter Mode Cipher Block Chaining Message Authentication Code (CCM); Broadcast/Multicast Integrity Protocol (BIP); Galois Message Authentication Code (GMAC); Message Integrity Code (MIC).

**Table 3:** Technical properties (top) and key performance indicators (bottom) of IIoT wireless technologies based on 802.15.4 2.4 GHz PHY layer and BLE.

	WirelessHART	ISA100.11a	BLE	802.15.4e TSCH
**Band**	2.4 GHz ISM	2.4 GHz ISM	2.4/5 GHz ISM	2.4 GHz ISM
**Bandwidth**	200 kHz–1.2 MHz	2 MHz	2 MHz	2 MHz/5 MHz
**Topology**	mesh	star/mesh/star-mesh	p2p/star/mesh	star, tree, mesh
**Deployment**	private	private	private	private
**MAC**	time sync., freq. hopping	TDMA / CSMA/CA (10–12 ms)	TDMA	TSCH
	TSMP (TDMA, 10ms)			(TDMA/CSMA/CA)
**Retransmissions**	yes	yes	yes	yes
**Reliability mechanisms**	ARQ, FHSS	ARQ, FHSS, DSSS	FHSS, 24-bit CRC,	DSSS/OQPSK
	DSSS, 32-bit MIC	32-to-128-bit MIC	32-bit MIC, FEC
**Range**	<1.5 km (225 m)	<1.5 km (100 m)	<100 m/<1000 m	<200 m
**Nodes per network**	30,000/hundreds per AP	unlimited/thousands per GW	unlimited	unlimited
**Data rate**	<250 kbps	<250 kbps	125 kbps/1 Mbps/2 Mbps	250 kbps
**Min. cycle time**	500 ms	500 ms	50 ms	20 ms

Automatic Repeat reQuest (ARQ); Frequency-Hopping Spread Spectrum (FHSS); Direct Sequence Spread Spectrum (DSSS); Gateway (GW); Offset QPSK (OQPSK); Message Integrity Code (MIC).

**Table 4:** Technology-specific parameters for energy consumption simulations.

	IEEE 802.15.4g	IEEE 802.11ah	LoRaWAN	NB-IoT
**Radio module**	Atmel AT86RF215		SEMTECH SX1272	uBlox SARA N210
**TX power (dBm)**	14		23	20
**Power (mA)**	28/62/6.28/0.03		11.2/125/0.0015/0.0001	46/220/6/0.003
**[RX/TX/idle/sleep]**				
**Technology-specific**	113 slots @ 40 ms per frame	4096 ms beacon interval	no repetitions	RRC: 10 s, DRX: 0 s,
**parameters**	2-FSK—100 kHz (50 kbps)	MCS 10 —1 MHz (150 kbps)	no ACK	PSM: TI s
	0.33% DIO and EB probability	1 RAW group, 1 slot		no repetitions
**Payload size**	104 bytes		12 bytes	
**Microcontroller**	ARM Cortex M3 @ 32 MHz (3.38 mA power consumption)			

Frequency Shift Keying (FSK); Enhanced Beacon (EB); DODAG Information Object (DIO); Destination Oriented Directed Acyclic Graph (DODAG); Modulation and Coding Scheme (MCS); Restricted Access Window (RAW); Radio Resource Control (RRC); Power Saving Mode (PSM); Transmission Interval (TI); Discontinuous Reception (DRX)
